# Hepatitis E Virus Seroprevalence in Austrian Adults: A Nationwide Cross-Sectional Study among Civilians and Military Professionals

**DOI:** 10.1371/journal.pone.0087669

**Published:** 2014-02-03

**Authors:** Heimo Lagler, Wolfgang Poeppl, Heidi Winkler, Harald Herkner, Angelus Faas, Gerhard Mooseder, Heinz Burgmann

**Affiliations:** 1 Department of Medicine I, Division of Infectious Diseases and Tropical Medicine, Medical University of Vienna, Vienna, Austria; 2 Department of Dermatology and Tropical Medicine, Military Hospital Vienna, Vienna, Austria; 3 Department of Emergency Medicine, Medical University of Vienna, Vienna, Austria; 4 Institute for Medical Support, Military Hospital Vienna, Vienna, Austria; The Australian National University, Australia

## Abstract

**Background:**

Hepatitis E Virus (HEV) infection is globally increasing. The present study was performed to investigate the HEV seroprevalence, exposure risks as well as occupational risks for military personnel in Austria, a Central European country.

**Methods and Findings:**

A nationwide cross-sectional seroprevalence study was performed in 997 healthy Austrian adults, professional soldiers and civilians. Routine laboratory and HEV specific antibodies were determined. In addition, epidemiological information on possible risk factors for exposure to HEV was obtained. The overall seropositivity for HEV antibodies was 14.3% and significantly increased with age. Seroprevalence was significantly higher among individuals with previous military employments abroad (21.4% vs. 9.9%) and among professional soldiers aged 30–39 years (20.2% vs. 7.3%). No association was found for private travel, occupational or private animal contact or regular outdoor activities. Individuals who tested positive for antibodies against HEV had significantly higher laboratory values regarding liver enzymes, lipid levels and blood fasting glucose.

**Conclusions:**

Exposure to HEV is common in Austria. Military employment abroad could be a potential risk factor for HEV infection. Further studies are required to investigate the significance of pathological laboratory results found among asymptomatic individuals previously exposed to HEV.

## Introduction

Hepatitis E virus (HEV) is a non-enveloped, single-stranded RNA virus belonging to the genus *Hepevirus* in the *Hepeviridae* family. HEV has initially been identified as an enterically transmitted “epidemic, non-A, non-B hepatitis” in the early 1980s in developing countries, while the viral genome was characterized one decade later [1], [2]. There is, however, evidence that worldwide human outbreaks of icteric illness due to HEV infections had already occurred more than 200 years ago, particularly in Europe [3]. Until recently, HEV infection was considered to be a travel-associated disease widely endemic in developing countries. In the last decade, however, HEV infection has increasingly been recognized as an emerging disease in both developing and industrialized countries.

HEV is currently classified into four major genotypes, which all belong to one serotype. These genotypes differ in terms of epidemiological distribution, clinical presentation and host species. Genotype 1 and 2 are endemic in developing countries and infect only humans, while no animal reservoir has been identified. Infection usually occurs by fecal-oral transmission and may result in hepatitis with fever, jaundice and nausea. In developed countries, these two genotypes are known as travel-associated HEV infection. By contrast, genotype 3 is endemic mainly in Europe and Northern America and genotype 4 in Asia [4]. Apart from human infection, genotypes 3 or 4 have also been isolated in several mammalian species, such as domestic pigs, wild boar, deer, and rodents, but also marine animals [5]. Infection in humans may result from consumption of raw or not well-cooked nutritional constituents or direct contact with infected animals [6], [7]. In addition, HEV has been detected in soil, river water, waste, as well as pig slurry, and may therefore be considered a zoonotic disease transmitted through exposure to contaminated environment [8]–[11]. Recently, additional HEV genotypes have been identified in farmed rabbits, rats and birds [12]. The relevance of these findings for human infection is yet unclear. Infection with HEV genotype 3 or 4 is usually asymptomatic or mild and patients will probably escape medical attention [13]. However, acute hepatitis may occur, and severe disease with rapid progression and liver-failure has been reported in pregnant women, immuno-compromised patients as well as patients with chronic underlying liver diseases [4].

Globally, the impact of HEV infections on public health has apparently been underestimated. In Europe, seroprevalences differ considerably, ranging from 0.26% in central Greece to 52.5% in South-Western France, but little information is available on exposure risks or potential high-risk groups [14], [15].

Since its discovery, several outbreaks of HEV infection have been reported among military personnel, particularly during or following activities in endemic areas [4], [16]–[19]. Thus, military personnel are considered an occupational high risk group for HEV infection. However, this assumption is based on case series and outbreak reports only, as no studies have been performed comparing exposure to HEV between professional soldiers and civilians.

Thus, the present study was initiated in order to investigate the seroprevalence of specific antibodies against HEV in (apparently) healthy adult individuals in Austria and to identify possible exposure factors for military personnel as well as civilians.

## Materials and Methods

### Subjects and study design

We have performed a nationwide cross-sectional study in healthy Austrian adults volunteering for military employments abroad. Before potential selection, all Austrian applicants (civilians as well as professional soldiers), have to undergo a standardized medical examination including routine laboratory investigations at the Military Hospital Vienna. The routinely performed laboratory investigations include erythrocyte sedimentation rate, total blood counts, aspartate aminotransferase (ASAT), alanin-aminotransferase (ALAT), gamma-glutamyl transferase (gGT), total bilirubin, cholesterol including low density lipoprotein (LDL), high-density lipoprotein (HDL), uric acid, serum creatinine and fasting glucose.

The study was approved by the Institutional Review Board of the Austrian armed forces and written informed consent was obtained from each participant before inclusion in the study.

For study purposes, an additional serum sample was obtained from each participant and stored at −20°C until qualitative testing for HEV specific IgG antibodies using an enzyme linked immunosorbent assay (ELISA, Fortress Diagnostics Limited, Antrim, United Kingdom) performed according to the manufacturer’s instructions. According to the package insert, this ELISA is especially suitable for HEV prevalence studies amongst larger populations, as 86% of 50 samples were still positive on re-testing ten years after confirmed HEV infection. This kit uses one long recombinant protein (PE2) of a Chinese strain belonging to genotype 1, and the sensitivity of this ELISA kit was 95% in samples from patients with PCR-confirmed HEV genotype 3 infection [20]. The ELISA Kit produced by Fortress, as used in this study, has given identical results in direct comparison to another assay produced by Wantai (Beijing Wantai Biological Pharmacy Enterprise Co., Ltd, China) [21]. The specificity for the ELISA produced by WANTAI was evaluated in >9000 persons and was shown to be 98.6% according to the package insert.

All samples were tested in duplicate. Each sample with an individual absorbance (OD) of a specimen/cut off (CO) value ≥0.9 was retested in duplicate and was considered positive only if the OD/CO value of both replicates was ≥1.0. The positive control was part of the kit provided by the manufacturer.

In addition, all participants were asked to complete an epidemiological questionnaire on demographic characteristics and potential risks of exposure (e.g. domestic and occupational animal contact, outdoor activities, holiday destinations abroad within the previous six months, previous military missions abroad).

### Statistics

In the absence of reliable estimates on HEV prevalence, we did not perform a formal sample size calculation. Instead, we used a convenient sample size of approximately 1000 individuals based on the available resources. With this sample, one can expect a 95% confidence interval (CI) from 8 to 12% assuming a prevalence of 10%. Data are presented as mean ± standard deviation (SD); categorical data are presented as absolute and relative frequencies. The Mann–Whitney U test, Chi-squared and Fisher’s exact test were used as appropriate for hypothesis testing to describe differences between negative and positive individuals. Prevalence of anti-HEV antibodies was described as relative frequency with exact 95% CIs. Odds ratios (OR) with exact 95% CIs were calculated using logistic regression models for identification of risk factors for seropositivity to HEV. MS Excel 2011 and Stata 11 for Mac (College Station, Tx) were used for data management and analysis. A two-sided P-value of <0.05 was considered statistically significant.

## Results

In total, 997 healthy Austrians aged between 18–59 years participated in the study from April to September 2009, with 980 (98.3%) being male and 17 (0.7%) female. The mean age of all individuals was 29±9.3 years; 407 (40.8%) participants were professional soldiers and 590 (59.2%) were civilians.

Overall, 143 sera (14.3%, 95% CI 11.5%–15.8%) showed a positive reaction for IgG antibodies against HEV indicating previous exposure. The detected OD/CO rates of the IgG HEV ELISA showed a clear bimodal distribution. In particular, the median OD/CO value was 6.1 (25% percentile 2.1, 75% percentile 14.6) in the 143 samples tested positive and 0.2 (25% percentile 0.1, 75% percentile 0.3) in the 860 negative samples.

The mean age of individuals who tested positive for the presence of HEV antibodies was 35.1±11.3 years versus 27.9±8.4 years for those with negative screening results (p<0.001). We found a significant trend for increasing seropositivity with increasing age (p for trend <0.001). Among individuals aged up to 19 years, the seroprevalence was 8.1% and increased to a seroprevalence rate of 57.5% (OR 15.46, 95% CI 5.71–41.82, p<0.001) among individuals aged 50–60 years (Table 1).

**Table 1:** Age and geographical distribution of 143 individuals seropositive to Hepatitis E virus among 997 healthy adults.

	Positives/Total (%)	Odds ratio (95% CI)	P value
**Age groups**			
18–19	7/87 (8.1%)	1 (reference)	–
20–29	51/544 (9.4%)	1.18 (0.52 to 2.70)	**0.69**
30–39	28/209 (13.4%)	1.78 (0.74 to 4.21)	**0.20**
40–49	33/117 (28.2%)	4.49 (1.88 to 10.73)	**0.001**
50–60	23/40 (57.5%)	15.46 (5.71 to 41.82)	**<0.001**
**Federal provinces of Austria**			
Vienna	16/128 (12.5%)	1 (reference)	–
Lower Austria	41/223 (18.4%)	1.58 (0.85 to 2.94)	**0.15**
Upper Austria	19/148 (12.8%)	1.03 (0.51 to 2.10)	**0.93**
Salzburg	7/63 (11.1%)	0.88 (0.34 to 2.25)	**0.78**
Tyrol	6/55 (10.9%)	0.86 (0.32 to 2.32)	**0.76**
Vorarlberg	1/19 (5.3%)	0.39 (0.05 to 3.11)	**0.37**
Burgenland	12/59 (20.3%)	1.79 (0.79 to 4.06)	**0.17**
Styria	19/167 (11.4%)	0.90 (0.44 to 1.83)	**0.77**
Carinthia	20/133 (15.0%)	1.24 (0.61 to 2.51)	**0.55**

Abbreviations: CI, confidence interval.

Concerning differences between the various provinces of Austria, seropositivity was lowest in the Western parts of Austria with seroprevalences of 5.3% in Vorarlberg, and increased to the East with a seroprevalence of 20.3% in Burgenland. These differences, however, were not statistically significant (p = 0.35; Table 1).

Univariate associations between potential risk factors for positive serology results are presented in Table 2. Specifically, there was no statistically significant difference in HEV seroprevalence between occupational animal contact and ownership of companion animals. No association was found for seropositivity against HEV virus and regular outdoor-activities.

**Table 2:** Risk factors for positive hepatitis E screening results.

Risk factors	Level	Positives/Total (%)	Odds ratio (95% CI)	P value
**Male**	yes	139/980 (14.2%)	0.77 (0.22 to 2.72)	0.72
	no	3/17 (17.7%)		
**Outdoor activities**	yes	113/795 (14.2%)	0.99 (0.64 to 1.54)	0.96
	no	29/202 (14.4%)		
**Professional soldier by age**				
18–19	yes	4/35 (11.4%)	2.11 (0.44 to 10.01)	0.35
	no	3/52 (5.8%)		
20–29	yes	19/190 (10.0%)	1.12 (0.62 to 2.03)	0.71
	no	32/354 (9.0%)		
30–39	yes	20/99 (20.2%)	3.23 (1.35 to 7.71)	0.008
	no	8/110 (7.3%)		
40–49	yes	10/57 (17.5%)	0.34 (0.15 to 0.81)	0.01
	no	23/60 (38.3%)		
50–60	yes	16/26 (61.5%)	1.60 (0.43 to 5.94)	0.48
	no	7/7 (50.0%)		
**Previous mission abroad**	yes	81/378 (21.4%)	2.49 (1.74 to 3.58)	<0.001
	no	61/619 (9.9%)		
Central or Northern Europe	yes	0/3 (0%)	n.a.	1
	no	81/375 (21.6%)		
Southern Europe	yes	57/285 (20.0%)	0.72 (0.42 to 1.24)	0.24
	no	24/93 (25.8%)		
Middle East	yes	27/129 (20.9%)	0.96 (0.57 to 1.61)	0.87
	no	54/249 (21.7%)		
Africa	yes	4/14 (29.0%)	1.49 (0.46 to 4.88)	0.51
	no	77/364 (21.2%)		
Asia	yes	7/18 (38.9%)	2.46 (0.92 to 6.56)	0.72
	no	74/360 (20.6%)		
**Private travel abroad**	yes	38/216 (17.6%)	1.39 (0.93 to 2.08)	0.12
	no	104/781 (13.3%)		
**Occupational animal contact**	yes	8/62 (12.9%)	0.89 (0.41 to 1.90)	
	no	134/935 (14.3%)		
Meat processing	yes	0/4 (0%)	n.a.	1
	no	142/993 (14.3%)		
Canine unit	yes	0/4 (0%)	n.a.	1
	no	142/993 (14.3%)		
Hunter	yes	1/3 (33.3%)	3.03 (0.28 to 33.58)	0.37
	no	141/994 (14.2%)		
Kitchen	yes	1/14 (7.1%)	0.46 (0.06 to 3.54)	0.46
	no	141/983 (14.3%)		
Agriculture	yes	5/23 (21.7%)	1.70 (0.62 to 4.65)	0.31
	no	137/974 (14.1%)		
Horses/rider	yes	1/5 (20%)	1.51 (0.17 to 13.60)	0.54
	no	141/992 (14.2%)		
**Companion animal**	yes	60/471 (12.7%)	0.79 (0.55 to 1.13)	0.21
	no	82/526 (15.6%)		
Amphibians	yes	0/1 (0%)	n.a.	1
	no	142/996 (14.3%)		
Fish-keeping	yes	1/7 (14.3%)	1.00 (0.12 to 8.40)	0.1
	no	141/990 (14.3%)		
Arthropods	yes	1/4 (25.0%)	2.01 (0.21 to 19.50)	0.55
	no	141/993 (14.2%)		
Rabbit	yes	4/22 (18.2%)	1.35 (0.45 to 4.04)	0.59
	no	138/975 (14.2%)		
Dog	yes	33/232 (14.2%)	1.00 (0.66 to 1.52)	1
	no	109/765 (14.3%)		
Cat	yes	31/300 (10.3%)	0.61 (0.40 to 0.93)	0.02
	no	111/697 (15.9%)		
Rodent	yes	3/18 (16.7%)	1.21 (0.35 to 4.23)	0.77
	no	139/979 (14.2%)		
Horse	yes	3/10 (30.0%)	2.62 (0.67 to 10.23)	0.17
	no	139/987 (14.1%)		
Reptile	yes	0/19 (0%)	n.a.	0.09
	no	142/978 (14.5%)		
Bird	yes	0/5 (0%)	n.a.	1
	no	142/992 (14.3%)		

Abbreviations: CI, confidence interval; n.a., not applicable.

Regarding military activities, the proportion of seropositive samples was significantly higher among professional soldiers aged 30–39 years when compared to civilians, but significantly lower among professional soldiers aged 40–49 years (p for interaction 0.0049; Table 2). Overall, 378 (37.9%) individuals had had at least one military employment abroad, and the seroprevalence was significantly higher in this cohort (21.4% vs. 9.9%, p<0.001). A positive correlation was also found with the number of previous military employments abroad (OR 1.26, 95% CI 1.14–1.39, p<0.001).

Concerning private travel abroad, 216 (21.7%) individuals reported at least one private travel abroad in the last six months. While no significant association was found (17.6% vs. 13.3%), the rate of seropositivity increased significantly with the number of private travel activities (OR 1.32, 95% CI 1.02–1.71, p = 0.04). Combining military employments abroad and private travel activities, a total of 486 (48.7%) individuals reported a history of having travelled abroad, and the seroprevalence was significantly higher in this group versus the cohort of “non-travellers” (19.3% vs. 9.4%; Table 2).

In terms of routine laboratory results, individuals who tested positive for antibodies against HEV had significantly higher values regarding liver enzymes, blood cholesterol and blood fasting glucose. The results in detail were as follows (including normal range): ALAT 32±18 U/l vs. 29±16 U/l, p = 0.01 (female <35 U/l, male <50 U/l); gGT 30±23 U/l vs. 24±16 U/l, p<0.001 (<60 U/l); cholesterol 202±42 mg/dl vs. 188±39 mg/dl, p<0.001 (<200 mg/dl); LDL cholesterol 134±36 mg/dl vs. 121±35 mg/dl, p<0.001 (<160 mg/dl); fasting glucose 98±9 mg/dl vs. 96±8 mg/dl, p = 0.01 (74–109 mg/dl).

## Discussion

In this first large Austrian study, the overall seroprevalence of 14.3% appears comparable to data obtained in healthy blood donors from other developed countries and identical HEV IgG ELISA. A seroprevalence of 21.8% was reported in 2009 for South-Western Switzerland, while the seroprevalences reported in South-Western England and the USA were 16% and 18.8%, respectively [20], [22], [23].

Of interest are older data reported for the year 1995, when a seroprevalence of only 2.3% was found among 1094 adult individuals in Vienna [24]. However, it is known that estimates of HEV seroprevalence are highly dependent on assay sensitivity and that assays with improved sensitivity result in higher seroprevalence rates [15], [20], [21], [23]. As the assay used in our present study is different from the one applied in the study from Vienna in 1995, one cannot interpret the difference as an increase in prevalence due to the higher sensitivity of our HEV IgG ELISA [20], [21].

In terms of geographical distribution within Austria, the lowest rate of 5.3% was found in individuals from the Western federal province Vorarlberg. In general, the present data suggested an increasing seroprevalence from Western to Eastern Austria. Unlike other viral hepatitis infections occurring in humans, HEV infection may also be considered a zoonosis and especially pigs are considered important reservoir animals. In Austria, the HEV genotype 3 has recently been detected in pigs [25]. Thus, a possible explanation for the geographic differences might be the distribution of industrialized pig farms in Austria, as the federal districts in the middle and Eastern parts of Austria where high seroprevalences were found (Burgenland, Lower Austria, Upper Austria and Styria) correspond to the regions of industrialized pig farming.

Interestingly, the data obtained from the present study revealed a distinct and significant increase of HEV-antibodies with age, with the seroprevalence being >55% in individuals aged 50–59 years. The nationwide seroprevalences in this age group is thus comparable to a hyperendemic area in South-west France [15]. Age dependent HEV seroprevalences, however, have also been reported in previous studies independent of the HEV ELISA assays used, although to our knowledge not all to this extent [18], [26], [27]. In Germany, the seroprevalence increased from approximately 6% among persons aged 20–29 to approximately 20% among persons 50–59 years of age [28].

Concerning occupational risk, HEV virus has been recognized as a potential threat to military forces since its discovery [4]. However, only a few studies have investigated the risk of HEV exposure for military personnel so far. From Nepal and Pakistan, seroprevalence rates of 30% among military personnel have been reported [16], [17]. Among 1500 US military service members deployed to Afghanistan, a low seroconversion rate of only 0.13% has recently been reported [18]. Among soldiers from Thailand being on active duty in the HEV-endemic areas in East Timor, Afghanistan, Burundi, and Iraq, the seroconversion rates were 1.9%, 4.6%, 4.6% and 3.9%, respectively [19]. Data directly comparing HEV exposure among soldiers and civilians, however, are not available. Among the study population used in our present series, a significant association for seropositivity and professional soldiers was found in individuals aged 30–39 years. Additionally, the present data indicate that previous military missions abroad (Table 2), as well as the number of previous military employments are risk factors for exposure to HEV. However, further studies are needed in order to assess the potential influence of repeated military missions in endemic regions abroad [18], [19], [29]. It has been suggested that military personnel employed in countries endemic for HEV may have increased exposure to contaminated water, contaminated food sources and respective sanitary conditions, which have been reported as risk factors for HEV transmission [18]. Although HEV infection is considered a travel-associated disease, private travel abroad in general was not associated with a higher risk of HEV exposure. However, seropositivity was significantly higher among frequent travellers with more than one trip abroad within the last six months before inclusion in the study.

Additional potential risk factors for HEV were evaluated in the present study, but no association between HEV-seropositivity and occupational animal contact, companion animals or outdoor activities could be found. One of the potential caveats, however, is the fact that the cohorts presenting with these specific risk factors are too small to allow for definitive conclusions.

In general, HEV infection is considered an emerging disease with a increasing prevalence worldwide. Fortunately, the great majority of infections among healthy individuals are mild and self-limiting and are apparently not associated with prolonged liver dysfunction. Of interest is the fact that significantly higher levels of liver enzymes, blood fatty acids and fasting glucose were detected among healthy individuals seropositive to HEV in the present study, even though they were still within the physiological range. This finding merits further investigations on the long-term medical relevance of exposure to HEV in healthy adults.

A possible limitation of the study is the fact that the population studied almost exclusively comprised of healthy male adults. However, outbreak reports as well as epidemiological studies have shown no significant difference concerning sex and exposure to HEV [28], [30], [31]. Thus, the male predominance in our study population does not appear to constitute a major confounding factor. Seroprevalences, however, are dependent on the sensitivity and specificity of the assay used in various studies. As a consequence, a comparison of seroprevalence studies performed with different assays should be avoided [15], [20], [21],^23^ and seroprevalence studies with different HEV assays are of limited significance. In addition, further confirmatory assays for HEV IgGs are currently not available.

In conclusion, our data demonstrate that exposure to HEV is a common phenomenon in the Austrian population studied. Military employments abroad and frequent travel activities could be potential risk factors. Further studies are warranted to investigate the significance of differences found concerning laboratory results among asymptomatic individuals previously exposed to HEV.
